# Neuroprotective Action of Humanin and Humanin Analogues: Research Findings and Perspectives

**DOI:** 10.3390/biology12121534

**Published:** 2023-12-16

**Authors:** Chrysoula-Evangelia Karachaliou, Evangelia Livaniou

**Affiliations:** Immunopeptide Chemistry Lab., Institute of Nuclear & Radiological Sciences & Technology, Energy & Safety, National Centre for Scientific Research “Demokritos”, P.O. Box 60037, 153 10 Agia Paraskevi, Greece; xrisak15@hotmail.com

**Keywords:** humanin, mitochondrial-derived peptides, humanin analogues, neuroprotective action, Alzheimer’s disease, neurodegenerative diseases

## Abstract

**Simple Summary:**

Humanin was discovered in 2001 as a biological factor with neuroprotective activity. This review article presents research results that have supported the neuroprotective action of humanin as well as of specific humanin analogues (most of which are synthetic) in various cellular and animal models; it also presents possible mechanisms of neuroprotective action and briefly discusses the prospects for exploiting the therapeutic potential of humanin and humanin analogues in the fight against neurodegenerative disorders.

**Abstract:**

Humanin is a 24-mer peptide first reported in the early 2000s as a new neuroprotective/cytoprotective factor rescuing neuronal cells from death induced by various Alzheimer’s disease-associated insults. Nowadays it is known that humanin belongs to the novel class of the so-called mitochondrial-derived peptides (which are encoded by mitochondrial DNA) and has been shown to exert beneficial cytoprotective effects in a series of in vitro and/or in vivo experimental models of human diseases, including not only neurodegenerative disorders but other human diseases as well (e.g., age-related macular degeneration, cardiovascular diseases, or diabetes mellitus). This review article is focused on the presentation of recent in vitro and in vivo research results associated with the neuroprotective action of humanin as well as of various, mainly synthetic, analogues of the peptide; moreover, the main mode(s)/mechanism(s) through which humanin and humanin analogues may exert in vitro and in vivo regarding neuroprotection have been reported. The prospects of humanin and humanin analogues to be further investigated in the frame of future research endeavors against neurodegenerative/neural diseases have also been briefly discussed.

## 1. Discovery of Humanin 

Humanin was discovered in 2001 as a 24-mer peptide that inhibited neuronal cell death induced by neurotoxic amyloid-β (Aβ) peptides and by mutants of familial Alzheimer’s disease (FAD) genes, i.e., APP amyloid precursor protein (APP), presenilin-1 (PS1), and presenilin-2 (PS2) [1,2,3]. More specifically, first humanin cDNA was discovered by functional expression screening of a cDNA library constructed with mRNAs obtained postmortem from the occipital lobe of a patient with Alzheimer’s disease (AD), i.e., a brain region that remains relatively intact in AD patients. AD is the most common neurodegenerative disorder characterized by deposition of Aβ plaques, neurofibrillary tangles of tau-protein, and massive neuronal cell death as well as by severe cognitive deficits and progressive memory loss, which make the patients’ daily life extremely hard. The term “humanin” (HN) was given to the novel neuroprotective peptide by Professor Nishimoto, at whose lab HN was discovered, to point out the potential of this peptide to bring back “humanity” in AD patients [4]. In 2003, two research groups [5,6] isolated HN, independently of each other and of Nishimoto’s group, by yeast two-hybrid screening as a peptide–ligand binding of (i) the insulin-like growth factor-binding protein-3 (IGFBP3), which is the major binding protein of IGF-I in circulation and also occurs intracellularly, and (ii) Bax, which is a proapoptotic protein of the Bcl-2 family. As reported, HN blocked the IGFBP3-induced cell death [6], while, through binding to Bax, it inhibited cell apoptosis [5]. Since the initial discovery and early studies, many groups worldwide have studied HN mainly in relation to neurodegenerative diseases but also other human diseases.

## 2. HN and HN Analogues with Biological Activity

The amino acid sequence of HN (MAPRGFSCLLLLTSEIDLPVKRRA, MW: 2687.26 Da) is characterized by three regions: a polar C-terminal (PVKRRA), a positively charged N-terminal (MAPR), and a central hydrophobic region (GFSCLLLLTSEIDL) [3,7,8]. The role of each amino acid within HN was investigated through systematic single-amino-acid substitution and the results could be summarized as follows: (i) L^9^, L^10^, and L^11^ residues, and especially L^10^, are important for the biological action of HN, i(i) L^9^, L^10^, and L^11^ and P^19^-V^20^ are critical for the extracellular secretion of the peptide, (iii) P^3^, S^7^, C^8^, L^9^, L^12^, T^13^, S^14^, and P^19^ are necessary for the neuroprotective action of HN, (iv) S^7^ and L^9^ are involved in self-dimerization of HN, which seems to be necessary for neuroprotection [8]. HN has been chemically synthesized in high overall yield following the solid-phase peptide synthesis strategy [9]. Early nuclear magnetic resonance (NMR) studies of HN in aqueous solution have revealed a flexible peptide, with definite turn points in its structure, which is free to interact with possible receptors/partners required for its action; in a less polar environment (30% TFE, 2,2,2-trifluoroethanol), HN adopts an α-helical structure (residues G^5^–L^18^) facilitating other specific interactions and/or membrane entry [10]. 

Shortly after the discovery of HN, an HN-like peptide was found in rat tissues and called rattin (RN); RN contains 24 residues that are highly homologous to those of HN along with 14 additional residues at its C-terminus and showed neuroprotective activity in primary neurons [11,12]. 

Not only parent HN but also some synthetic analogues of the peptide have been proved highly active; thus, HNG, in which the serine residue at position 14 was substituted with glycine, exhibited a 1000-fold higher neuroprotective activity than HN [1,2,3]. Moreover, HNG exhibited a 500-fold higher rescue activity in cortical neurons which led to apoptosis by a prion peptide [13]. The in vivo effects of synthetic HNG were evaluated shortly after the discovery of parent HN; more specifically, the experimentally induced (by scopolamine-HBr) impairment of spontaneous alternation behavior of mice in a Y-maze was reversed by intracerebroventicular administration of HNG [14]. As further shown, chronic intraperitoneal (i.p.) administration of HNG could significantly ameliorate spatial learning and memory deficits and decrease accumulation of Aβ plaque and concentration of insoluble Aβ by reducing neuroinflammatory responses in middle-aged APPswe/PS1dE9 mice [15]. Shortly earlier, Niikura et al. [16] reported similar in vivo findings in APPswe, tau_P310L_, and PS1_M146V_ triple transgenic mice (3xTg-AD) after chronic intranasal administration (i.n.) of HNG [16]. As revealed with subsequent studies, intrahippocampal administration of HNG protected from spatial learning and memory deficits induced in rats by Aβ(1–42) and Aβ(31–35) [17]. In further studies, i.p. injection of HNG ameliorated object memory deficit induced in mice by diazepam [18], while it also ameliorated the effects of the demyelinating agent cuprizone on object recognition memory and neuroinflammation in mice [19]. NMR structural studies of HNG in an aqueous solution have revealed a flexible peptide, showing higher flexibility in comparison with parent HN. In a less polar environment (30% TFE), an α-helical structure has been identified (residues F^6^–T^13^). Flexibility has been considered an important structural feature, since it may facilitate interactions with various functional partners in the neuroprotection pathways [20].

In addition to HNG, an HN analogue bearing the D-form of the serine residue at position 14 (HN-D-Ser14) has been described [21]. More specifically, the cytoprotective activity of HN has been significantly increased by optical isomerization of the serine residue at position 14 from L- to D-form, but details of a putative in vivo mechanism that might lead to this transformation remain unclear. In a subsequent study, the differences in biological activity among commercially available HN, HNG, and HN-D-Ser14 were compared by employing Aβ as a binding partner of the peptides. As reported, HN-D-Ser14 could act against fibrillation of Aβ showing significantly higher binding affinity for Aβ than those of both parent HN and HNG. In addition, the solution structure of HN-D-Ser14 was studied with NMR and a significant rearrangement in the conformation of parent HN has been revealed, which might contribute to the higher binding affinity of HN-D-Ser14 for Aβ. Moreover, by the NMR studies an Aβ-binding site was localized on HN-D-Ser14 [22].

Various other HN analogues, with different and/or additional to G^14^ amino acid substitutions, have been reported. For instance, substitutions of R^4^ and F^6^ with alanine in HNG have been introduced, aiming at the prevention of possible proteolysis by trypsin and chymotrypsin, leading to the potent analogue, AGA-HNG [23]. 

Another HN analogue, HNGF6A, has been produced by amino acid substitution at position 6 (i.e., A^6^ for F^6^), and position 14 (i.e., G^14^ for S^14^) and exhibited therapeutic potential in various experimental models of human diseases, such as diabetes [24]. HNGF6A could not bind to IGFBP3 and showed distinct pharmacokinetic characteristics when administered to male rodents [7,25]. As recently shown, HNGF6A exerted cytoprotection from oxidative-stress-induced apoptosis and osteogenesis promotion in MC3T3-E1 cells [26]. 

In addition to 24-mer HN analogues, various other analogues, shorter or longer than parent HN, have been described. Thus, as reported previously, the HN fragment that is necessary and adequate for exerting neuroprotection is the sequence from P^3^ to P^19^, composed of 17 amino acid residues (HN17); moreover, alanine substitutions of R^4^ and F^6^ along with arginine substitution of C^8^ in HN17 have led to the highly potent neuroprotective HN analogue AGA-(C8R)HNG17, as shown in various in vitro and in vivo experimental models [23,27].

Colivelin (CL) is another synthetic HN analogue, which bears the so-called activity-dependent neurotrophic factor (amino acid sequence: SALLRSIPA) chemically coupled to the N-terminus of AGA-(C8R)HNG17; CL has exhibited neuroprotective activity against cell death induced by AD causative genes and Aβ peptide even at very low concentrations (100 fM–10 pM). Moreover, CL has been reported to ameliorate Aβ-induced impairment in special memory and synaptic plasticity when administered intrahippocampally in normal rats, as well as in learning and memory deficits induced in APP/PS1 mice after chronic i.n. administration [23,28,29]. Various analogues of HN/HN-D-Ser14 and CL were synthesized and tested for their ability to exert significant in vivo neuroprotective/antiamnesic effects via reversing memory impairment induced in rats by the potent anticholinergic agent 3-quinuclidinyl benzilate (QNB); some of these analogues contained the non-natural amino acid *tert*leucine (Tle), attempting to decrease proteolysis and thus increase in vivo stability. As revealed, CL and its synthetic analogue des-Leu-CL showed the highest activity, since both peptides, when administered i.p., could prevent the QNB-induced deterioration of the behavior of rats in a T-maze test [30]. The development of diverse derivatives of HN and CL was also described, including derivatives bearing a biotin- or fluorescein-isothiocyanate moiety as well as the ^99m^Tc-chelating unit *dimethyl*GSC or a tyrosine residue that could be ^125^I-radioiodinated; the molecular probes developed were subsequently used for the investigation of the mode of action of CL and HN [31,32,33]. 

Another analogue of HN, tHN-C3, was developed by employing molecular biology methods. This HN analogue contained a transducible sequence based on the human transcription factor Hph-1, which would presumably cross biological barriers/membranes, along with an extended caspase-3 cleavage sequence (KKRLGLPGDEVD), which would enable specific inhibition of caspase-3. tHN-C3 prevented neuronal cell death induced by H_2_O_2_ or soluble Aβ(1-42) via Bax binding; moreover, tHN-C3 could successfully prevent neuronal cell death and infiltration of inflammatory cells in the brain, while it improved cognitive memory in Tg2576 mice and in the rat middle cerebral artery occlusion model of stroke. Interestingly, the effectiveness of tHN-C3 was comparable to that of the approved AD treatment drug Aricept [34].

Two novel synthetic HN analogues, i.e., the so-called HUJInin and c(D-Ser14-HN), were reported. HUJInin contains the potent HN analogue AGA-(C8R)HNG17, along with the so-called NAP peptide or “davundetide” (amino acid sequence: NAPVSIPQ), which was identified as the smallest active sequence of the activity-dependent neuroprotective protein, elongated by an N-terminally coupled tyrosine residue (YNAPVSIPQ). On the other hand, c(D-Ser14-HN) is a cyclic peptide specifically designed to achieve high biological potency by exploiting earlier revealed structural features of HN-D-Ser14 [22]. Both analogues were synthesized with solid-phase peptide synthesis and exhibited neuroprotective (and myoprotective) actions in neuronal (and myoblast) cell cultures. Their neuroprotective activity was evaluated in PC12 and SH-SY5Y neuronal cell models and found to be high and dose dependent [35].

A commercially available hybrid peptide, HNSS, which is composed of HNG and the antioxidant mitochondria-targeting peptide SS31 elongated by two C-terminal glycine residues (amino acid sequence: R(d)-Dmt-KFGG, where Dmt is 2′,6′-dimethyl tyrosine), has been recently evaluated. As reported, HNSS treatment through appropriate administration effectively recovered mitochondrial function, decreased Aβ deposition and tau hyperphosphorylation, rescued cholinergic neuron loss, and ameliorated cognitive deficit in 3xTg-AD mice [36]. 

The above-described HN analogues are presented in Table 1.

## 3. HN and HN-like Mitochondrial-Derived Peptides

HN contains less than 50 amino acid residues and can thus be considered a short peptide rather than a protein [37]. As is now well established, HN belongs to a wide class of peptides known as “mitochondrial-derived peptides” (MDPs). Functional MDPs are encoded by “small open reading frames” (sORFs, ≤300 nucleotides) located in the mitochondrial DNA. HN, which is transcribed from the mitochondrial genome at the 16S ribosomal RNA locus (MTRNR2 gene), is either stored in the mitochondrion or secreted into the cytoplasm [38]. The precise mechanism of transcription and translation of HN remains mostly obscure. The well-known 24-mer peptide appears to be present in cell cytoplasm, while a 21-mer variant appears to remain within mitochondria. These two forms of intracellular HN share the same domains and both seem to be functional [39]. In addition to humans, HN genes have been identified in many other species, in which pseudogenization of the HN gene often occurs. For instance, in mice, HN-encoding *Mtrnr2* is a mitochondrial pseudogene, and the HN-like peptide is encoded by the nuclear *Gm20594* gene [40]. Overall, the mitochondrial form of HN is considered as highly conserved across many species ranging from primates to nematodes, which is indicative of an ancient origin [38,41,42]. Interestingly, according to a recently published article, the 21-mer variant of HN is highly specific to mammals, while HNG might be present among avian HN sequences [43].

In addition to HN, which is considered the first mitochondrial sORF-encoded peptide with known biological activity, MDPs include small HN-like peptides 1–6 (SHLPs 1–6), MOTS-c, SHMOOSE, and Gau [44,45,46]. SHLPs 1–6 comprise a class of rather recently discovered peptides, the functions of at least some of which are similar to those of HN and are currently under investigation [47,48]. As recently reported, SHLP2 has shown potent metabolic benefits through its activity on pro-opiomelanocortin neurons in the hypothalamus of the central nervous system [49]. Many other currently uncharacterized MDPs may exist [45]. 

## 4. Recent Findings on the Therapeutic Potential of HN and HN Analogues against Neurodegenerative/Neural/Brain Disorders

Historically, HN has been associated with the most common neurodegenerative disease, AD, as a peptide protecting against AD-specific neurotoxicity induced by specific insults. Parkinson’s disease (PD) is another common neurodegenerative disease with several motor symptoms, such as bradykinesia, resting tremors, and postural instability, which has become a serious burden worldwide. Some studies have reported the central role of mitochondrial dysfunction in PD and recent research has demonstrated that HN might be considered an important approach for developing effective treatment strategies against PD [50]. Thus, Kim et al. [50] have recently demonstrated the neuroprotective effects of HN treatment in in vitro and ex vivo/in vivo PD models, which seem to promote mitochondrial biogenesis mainly via the PI3K/AKT signaling pathway. As reported, i.n. delivery of HN has promoted mitochondrial function, stimulated gene expression of HN itself, and exhibited beneficial effects against neurodegeneration, which allowed the researchers to propose HN as a lead molecule for the development of novel anti-PD therapeutics [50]. 

Some other in vitro and/or in vivo research findings of the last few years that refer to the therapeutic potential of HN and HN analogues in neurodegenerative/neural/brain disorders are presented below: 

Qian et al. [36] have reported the development of the hybrid peptide HNSS, which is an HN analogue composed of HNG and the antioxidant peptide SS31 (Table 1). The authors have developed a suitable peptide delivery system based on nanoparticles of citraconylation-modified poly(ethylene glycol)-poly(trimethylene carbonate) polymer (PEG-PTMC(Cit)), which could be loaded with HNSS through electrostatic interactions. In addition, taking advantage of the overexpression of fibroblast growth factor receptor 1 (FGFR1) in both the blood–brain barrier (BBB) and cholinergic neurons, the authors have added an FGFR1 ligand (FGL peptide), to the nanoparticle delivery system (FGL-NP(Cit)/HNSS), thus achieving increased brain accumulation with favorable distribution into cholinergic neurons at the diseased region. As reported, intravenous (i.v.) administration of FGL-NP(Cit)/HNSS in 3xTg-AD mice has reversed mitochondrial dysfunction via the PGC-1α and STAT3 pathways, inhibited Aβ deposition and tau hyperphosphorylation, and ameliorated memory deficits and cholinergic neuronal damage [36].

Jung et al. [51] have reported that HN could improve cognition deficits in male mice with induced intracerebral hemorrhage (ICH). HN, either (i) secreted from astrocytes, (ii) transferred within intact mitochondria released by astrocytes, or (iii) exogenously injected as a synthetic peptide, could lead to a “reparative” microglial phenotype, enhance phagocytic activity against red blood cells, and reduce proinflammatory responses. Overall, astrocytic mitochondrial-derived HN has been suggested to act as a beneficial secretory factor in ICH, while restoration of HN levels in the injured brain through exogenously administered HN could represent a therapeutic approach for ICH [51]. In a relevant study, Tashiro et al. [52] have reported that ICH is associated with increased superoxide generation, increased oxidative damage, and loss of the mitochondrial enzyme manganese superoxide dismutase (Mn-SOD). The detrimental effects of ICH were reversed by i.v. transplantation of astrocytic mitochondria, which are known to contain HN, while exogenously administered HN could simulate the effect of mitochondria transfer on detrimental effects in ICH-like injury; HN could also restore neurite extension inhibited by ICH in cultured neurons through increases in the expression of the synaptogenesis-related proteins synapsin 1 and PSD-95 [52]. 

Arneson et al. [53] have reported that the HN-encoding *mtRnr2* gene may be considered a potential therapeutic target for mild traumatic brain injury (mTBI), due to its extensive dysregulation in mTBI. As further shown, treatment of a murine mTBI model with HNG reversed cognitive deterioration caused by mTBI, probably via restoration of metabolic pathways within astrocytes [53].

Ikegawa et al. [54] have reported that the hippocampal acetylcholine (ACh) levels increase in mice after i.p. injection of HNG, which seemed to improve the object memory of mice. Moreover, in PC12 cells, HNG enhanced ACh-induced dopamine release. These in vitro and in vivo findings indicate that HN/HNG could directly enhance exocytosis in neurons and consequently improve cognitive effectiveness, which is a newly described physiological role for HN peptides [54].

Zhao et al. [55] have investigated the neuroprotective effects of synthetic HN on neurotoxicities induced by calyculin A (CA), an inhibitor of protein phosphatases PP2A and PP1, in cortical neurons. As revealed, incubation of cultured cortical neurons with HN prior to their treatment with neurotoxic CA could preserve neuronal cell viability, decrease oxidative stress, and preserve the activity of PP2A, while it also seemed to block hyperphosphorylation of tau protein [55]. 

Gilon et al. [35] have examined the neuroprotective (as well as myoprotective) effects of two novel synthetic HN analogues, HUJInin and c(D-Ser14-HN) (Table 1). More specifically, PC12 and SH-SY5Y neuronal cells, in which neurotoxicity had been induced with oxygen–glucose deprivation (OGD) and subsequent reoxygenation (R), were employed as cellular models. HUJInin and c(D-Ser14-HN) exerted significant dose-dependent in vitro neuroprotection, which was associated with decreases in OGD-induced Erk 1/2 phosphorylation, stimulation of AKT phosphorylation, and improvement of mitochondrial functions. Based on these findings, HUJInin and c(D-Ser14-HN) have been proposed as new lead molecules for the eventual development of novel drugs against stroke [35].

Gurunathan et al. [56] have demonstrated, through extensive in vitro studies, that HN could protect SH-SY5Y neuroblastoma cells against neurotoxicity induced by silver nanoparticles.

Zhao et al. [57] have investigated the neuroprotective effects of CL on ischemic brain injury, by employing mice with experimentally induced transient focal cerebral ischemia and reperfusion, along with potential mechanisms underlying putative protective effects. As revealed, CL administration could decrease neurological deficits and infarct lesions and inhibit axonal damage and neuronal death in brain tissue. Moreover, CL has been reported to induce antiapoptotic genes and activate JAK/STAT3 signaling [57].

Han et al. [58] have reported that i.p. administration of HNG could improve the learning ability and memory in APP/PS1 transgenic mice; as proposed, improvement has possibly been achieved through the regulation of IRS-1/mTOR insulin signaling in the hippocampus and the subsequent increase in the activity of autophagy and decrease in Aβ deposition in the brain [58].

Yen et al. [59] have demonstrated that HNG had neuroprotective effects in human SH-SY5Y neuroblastoma cells treated with Aβ, while i.p. administration of HNG could improve cognition in aged (>18-month-old) female mice, possibly through decreases in microglial activation and systemic inflammation; moreover, administration of HNG to female mice (18 months of age) was able to increase healthspan as shown by evaluating a series of different parameters [59]. 

Peng et al. [60] have reported that HNG could ameliorate cerebral infarction and suppress the production of TNF-α, IL-1β, IL-6, and MCP-1 cytokines in a middle cerebral artery occlusion (MCAO) stroke model developed in mice. Moreover, HNG could inhibit the expression of vascular adhesion molecules such as VCAM-1 and ICAM-1 in the cortex tissue. In mouse brain endothelial bEnd.3 cells, HNG could support cell survival under oxygen deprivation (OGD) conditions and suppress production of ROS as well as specific cytokines and vascular adhesion molecules induced by OGD. As reported, HNG exerts its effects by inhibiting the IKKα factor involved in the NF-κB pathway, activating IκBα and promoting accumulation of p65 in the nucleus [60]. 

The above research findings have been summarized in Table 2.

## 5. Therapeutic Potential of HN and HN Analogues against Various Other Disorders

According to accumulated in vitro and in vivo findings, treatment with HN and HN analogues has been considered as potentially beneficial in specific experimental models of disease, especially of various age-related diseases [44,61,62,63], such as age-related macular degeneration (AMD) [64,65,66]. In a general concept, HN and HN analogues may have a series of potential therapeutic applications in various human diseases, since HN plays an important role in the human body and seems to protect against several pathological conditions and symptoms. For instance, HN can help reduce inflammation and oxidative stress, thus interfering with the development of cardiovascular and autoimmune diseases. HN may also help mitochondria to maintain proper functioning; dysfunction of mitochondria can contribute to a series of health problems, associated, e.g., with metabolic disorders, such as diabetes and obesity, and this is in line with reports showing that HN may improve glucose metabolism and insulin sensitivity in animal models. The antiapoptotic activities of HN have been considered as a potential therapeutic target in the treatment of male infertility. Moreover, the roles of HN in the skeletal system are emerging, where it appears to be involved in the regulation of osteoclasts, osteoblasts, and chondrocytes [39,44,61,67,68,69,70,71,72]. The mechanisms behind the potentially beneficial effects of HN and HN analogues in different diseases are slowly being elucidated and appear to mainly be associated with the broad cytoprotective activity of HN.

Interestingly, HN and HN analogues have been proposed to present therapeutic potential against cancer, but the role of HN in tumorigenesis remains controversial [73]. For instance, in a study employing a male mouse model with pulmonary melanoma metastases, HNG has been reported to protect healthy male germ cells and leukocytes against cyclophosphamide, while it induced apoptosis in tumor cells and sensitized tumor suppression [74]. Moreover, in mice with human neuroblastoma or medulloblastoma tumor xenografts, HNG has been shown to prevent bone growth impairment caused by bortezomib as an undesirable side-effect, without interfering with the desired anticancer activity of bortezomib; this beneficial effect has been attributed to reduced angiogenesis and increased tumor cell apoptosis [75,76]. However, parent HN was initially reported as a potential oncopeptide [77], while contradictory findings concerning its role in cancer progression have appeared in the literature. Thus, a recent study has reported that HN might be tumor promoting in triple-negative breast cancer, because HN antagonizes the proapoptotic Bax [78], while silencing of HN expression has been proposed as a therapeutic approach to treat pituitary tumors [79]. Overall, further thorough studies are needed to clarify the effects of HN on different cancer cells [39,80].

## 6. Possible Mode(s) of Neuroprotective/Cytoprotective Action of HN and HN Analogues

**Figure 1:**
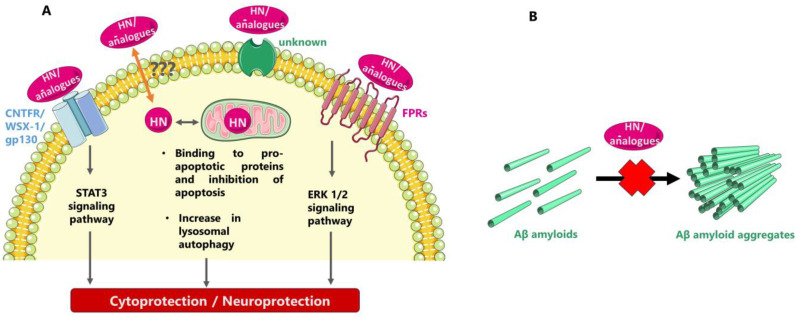
Possible modes of neuroprotective/cytoprotective action of HN and HN analogues. (**A**) Triggering of intracellular cell rescue mechanisms after interaction with cell membrane HN receptors and/or specific intracellular HN partners (the symbol “???” represents unknown putative mechanisms of secretion and/or entry into cells). (**B**) Inhibiting the formation of toxic Aβ fibrils in the extracellular space.

HN has been shown to exert both intra- and extracellular actions through multiple partners, some of which might be yet unknown. After interacting with proapoptotic proteins, such as IGFBP3 and Bax, HN intracellularly prevents apoptotic cell death, as initially shown; also, as later demonstrated HN may be localized on the lysosomal membrane and increase autophagy, which could also confer cytoprotection by directing various oxidized proteins to the lysosomes for degradation. Extracellularly, HN and HN analogues may interact with cell surface receptors, thus regulating cell survival and other important cellular processes. Inhibiting formation of toxic Aβ fibrils has also been proposed as a mode of the neuroprotective/cytoprotective action of HN and HN analogues (Figure 1).

Concerning its intracellular partners, HN was initially found to interact with the proapoptotic protein IGFBP3 and participate in associated cell survival pathways [6,39]. Thus, through its interaction with the C-terminal domain of IGFBP3, HN interferes with the binding of importin-β to IGFBP3, thereby suppressing IGFBP3-mediated apoptosis [81]. HN has also been reported to interact with and inhibit the action of the proapoptotic protein Bax, a member of the Bcl-2 family of proteins which regulate intrinsic or mitochondrial programmed cell death. HN could interact with other members of the Bcl-2 family, such as Bid, and regulate their translocation to suppress the production of apoptosomes and promote the expression of caspase-3 in mitochondria [5,39,82]. Recent studies have revealed further insight into the role of HN in mitochondria-dependent apoptotic effects and HN has been found to inhibit the mitochondrial membrane association and oligomerization of Bax and Bid proteins and to prevent Bod activation by sequestering it in fibers [83,84]. Additionally, HN has been reported to localize in the lysosomal membrane surface and increase the activation of chaperone-mediated autophagy; thus, HN confers cytoprotection by directing oxidized proteins to the lysosomes for degradation. HN-induced autophagy could reduce the accumulation of harmful misfolded proteins in skeletal muscle of mice [61,85,86,87]. 

The intracellular roles of HN were previously summarized by Popov et al. [70] as follows: (i) involvement in the retrograde signaling pathway from the mitochondria to the nucleus, (ii) participation in cell survival mechanisms as a stress responsive, cell survival factor acting against oxidative stressors by activation of the chaperone-mediated autophagy pathway, (iii) protection from cellular death by inhibiting the generation of reactive oxygen species (ROS), upregulation of mitochondrial glutathione (GSH) and activation of caspase-3 and caspase-4, (iv) defense against endoplasmic reticulum stress through restoration of the depleted mitochondrial GSH levels. 

Concerning the extracellular/membrane-bound partners of HN, two types of receptors have been reported so far for the peptide: a heterotrimeric receptor “complex” composed of ciliary neurotrophic factor receptor α, the cytokine receptor WSX-1, and the transmembrane gp130 (CNTFR/WSX-1/gp130), which conveys signals via the STAT3 signaling pathway, and the formyl peptide receptors (FPRs), which convey signals via the ERK 1/2 signaling path [38,39,88,89,90,91,92,93]. Moreover, studies in a rat model have shown that HN could rescue cortical neurons from N-methyl-D-aspartate (NMDA)-mediated neurotoxicity, but not through direct interaction with the NMDA receptor [94,95]; instead, HN seems to act through its interaction with the gp130 receptor and, more specifically, as shown with computational modeling, with the domain-4 and -5 of the gp130 receptor [96]. 

As recently reported, regulating the exocytosis of neurotransmitters may be a novel role of HN/HNG associated with the effects of HN and HN analogues on brain functions in health and disease [54]. Moreover, as recently demonstrated, HN treatment can induce mitochondrial biogenesis, especially by activating the PI3K/AKT signaling pathway, and thus stimulate expression of mitochondrial genes including that encoding HN itself [50].

Another approach to explain the neuroprotective/cytoprotective role of HN was through interaction of the peptide with neurotoxic Aβ [97]. Thus, shortly after the first articles reporting its discovery, HN was identified to interact with Aβ, being capable of transforming fibrillar Aβ(1-40) into an amorphous peptide [98]. Later, putative binding epitopes between HN and Aβ(1–40) were reported, as identified with affinity mass spectrometry and molecular modeling studies and verified with ELISA [99]. Later on, the in vitro interactions between HN and CL with Aβ(1-40) were studied through in-house-developed ELISA-type assays, in which HN-biotin and CL-biotin derivatives appeared to directly interact with increasing concentrations of Aβ(1-40) in a dose-dependent manner [33]. Moreover, as already mentioned an Aβ-binding site was localized on HN-D-Ser14 with NMR studies [22]. Another mechanism of HN-mediated protection against Aβ has been proposed to be the competition between HN and Aβ for binding to FPRs [88]. It might be of interest to note here that the HN analogue HNG along with the SHLP2 peptide have been reported to biophysically interact in vitro with the misfolded amyloid seeds of the islet amyloid polypeptide (IAPP, an intrinsically disordered amyloid protein that is co-expressed with insulin in pancreatic β-cells), via a chaperone-like mechanism; this interaction may regulate pancreatic β-cell amyloids and thus contribute to a cytoprotective action [100,101].

Although the molecular mechanisms behind the neuroprotective/cytoprotective actions of HN and HN analogues have been thoroughly investigated, the possibility that more than the so far described ones might exist cannot be excluded. 

## 7. Routes/Formats of Exogenous Administration of HN and HN Analogues

HN and HN analogues have been exogenously administered to normal animals and animal models of neurodegenerative/neural disorders as plain peptides through various routes, i.e., in a few cases intracerebroventicularly [14] or intrahippocampally [29] and more often i.p. [18,19,30,102] or i.n. [16]. As recently reported, synthetic HN that has been administered i.n. in an animal model of PD seems to enter the brain mainly through the perivascular space of the trigeminal nerve in a similar way to that of drugs bypassing the BBB [50]. Moreover, a fused “transducible” HN analogue (tHN-C3, Table 1) bearing, in addition to the HN-based sequence, a special moiety that could allow bypassing/crossing biological barriers was previously reported [34].

A more sophisticated approach to successfully pass across biological barriers, such as the BBB, and reach the target neuronal cells is to administer HN or HN analogues through a suitable delivery system, which would, in addition, minimize premature peptide degradation. Qian et al. [36] have recently developed a specific delivery system and used it for the i.v. administration of the hybrid peptide HNSS (Table 1) to 3xTg-AD mice. The system developed has been based on the so-called FGL peptide as well as on nanoparticles composed of citraconylation-modified poly(ethylene glycol)-poly(trimethylene carbonate) polymer (PEG-PTMC(Cit)). FGL is a neural cell adhesion molecule mimetic peptide that can be bound to fibroblast growth factor receptor 1 (FGFR1), highly expressed in the BBB and cholinergic neurons, with high affinity; the authors have used FGL-modified nanoparticles loaded with the HN analogue HNSS. The finally prepared nanoparticles have shown desirable HNSS-loading capacity, increased cholinergic neuron targetability, and accelerated intracellular HNSS release; moreover, HNSS has been self-directed to the mitochondria (through its SS31 moiety, Table 1), thus leading to a successful therapeutic outcome [36]. Previously, HNG incorporated into a specific delivery system was administered i.v. to mice and seemed to effectively reach brain cells; the delivery system employed was based on self-assembled poly(ethyleneglycol)poly (D,L-lactic-co-glycolic acid) polymersomes conjugated with lactoferrin [103,104]. 

In addition to issues associated with permeability through biological barriers, exogenous administration of HN and HN analogues has been challenging due to instability issues as well. Thus, to overcome low stability of HNG, because of the tendency of the peptide to rapidly degrade or oxidize, a special stabilization solution (called MO formulation) has been developed, which could stabilize the HNG peptide structure at both 4 °C and 37 °C for up to 28 days [105]. Stability issues have also been reported for radioiodinated (^125^I) HN derivatives administered to female mice via the tail vein, which probably prevented solid localization of specific in vivo targets for HN in subsequent biodistribution studies [31].

## 8. HN Levels in Health, Neurodegenerative Disorders, and Aging

According to the early articles describing its discovery, HN can be secreted into the blood circulation and transported to target cells [1,2,3,8]. Many subsequent studies have reported that HN, along with several other MDPs, is normally expressed in various organs, including heart, kidney, liver, sperm and testes, skeletal muscle, and brain as well as in specific cell types, such as glial cells, Leydig cells, and macrophages, while HN has also been found in human plasma, cerebrospinal fluid, and seminal and follicular fluid [7,39,54,106,107]. Nevertheless, it is still unclear which are the secretory mechanisms and which tissues contribute to the circulating pool of this peptide, while entry into cells is even more obscure [61]. As recently reported, HN could be released in the form of exosomes from SH-SY5Y cells, which might contribute to intercellular or intertissue signaling [108].

HN levels seem to be associated with several human diseases, including neurodegenerative disorders. Thus, in a recently published study, Salemi et al. [109] have evaluated HN mRNA levels through qRT-PCR analysis in peripheral blood mononuclear cells (PBMCs) of patients with PD and compared them with those found in PBMCs of control individuals. As shown, increased HN mRNA levels were measured in PD samples compared to controls. These findings demonstrate the tendency of mitochondria to overexpress mRNA in PD, which might be associated with a cellular attempt to ameliorate apoptotic damage occurring in PD patients. The authors have also proposed that HN might be useful as a marker for a better diagnosis and presumably prognosis of PD, also showing a therapeutic potential for treating PD [109]. It should be noted, however, that in another recent study no significant differences have been observed in circulating HN levels of PD patients as compared with control individuals [50]. On the other hand, Yen et al. [110] have reported decreased HN levels in CSF samples of patients with Alzheimer’s disease. Moreover, Xu et al. [111] have reported that hyperbaric oxygen (HBO) treatment might exert neuroprotective effects in vascular dementia by elevating the serum HN levels. More specifically, HBO treatment has been reported to increase serum HN levels, and HN levels have in turn been shown to positively correlate with the Mini-Mental State Examination scores employed to assess patients’ cognitive function [111]. 

Accumulated literature information indicates that decreased HN levels may be associated with cognitive deficits developed during aging and several research groups have reported a decrease in HN plasma levels with age in mice, monkeys, and humans, while other researchers have investigated the beneficial effects of HN on cognitive deficits [7,24,54,63,110,112]. HN levels have specifically been associated with postmenopause cognition decline. Thus, Zarate et al. [113] have reported that astroglial functional and morphological alterations induced by chronic ovariectomy in a menopause rat model resemble an aging phenotype and could affect astroglial support to neuronal function through alteration of synaptic connectivity and functionality. As measured with real-time quantitative polymerase chain reaction (RT-qPCR), decreased astroglial-derived HN levels have been found in the ovariectomized menopause rat model, which may be associated with and be indicative of an underlying mechanism for synaptic dysfunction and cognitive decline after menopause [113]. In addition, a specific single-nucleotide polymorphism (rs2854128) in the HN-coding region of the mitochondrial genome has been discovered, which is associated with a decrease in circulating HN levels and with accelerated cognitive aging in African Americans, a finding which seems to be race specific [59]. 

On the other hand, it should be noted that, according to other studies, plasma levels of HN have been positively correlated with age in humans, becoming maximal in centenarians, which seems to contradict the previously described findings [114]. The circulating HN levels have also been measured in centenarians’ children, who are believed to have a greater chance of becoming centenarians themselves, and found to be higher in comparison with age-matched controls; in addition, HN levels have been found to be stable in the naked mole-rat, a model of negligible senescence [63,110]. Overall, although HN levels have been associated with a series of age-related deficits, such as cognition decline, more research is required to completely elucidate this putative relationship. 

HN has been determined at both the peptide and mRNA levels. Circulating HN peptide concentrations were measured by in-house-developed ELISAs [24,25,110,112] or commercially available ELISA kits [114], while HN levels were sometimes semi-quantitatively assessed with Western blotting; the results obtained are therefore substantially dependent on the specificity of the HN antibodies used [40]. On the other hand, detection of HN at the mRNA level can also be a target of concern because of the unusual genomic structure of MDPs [40].

## 9. Conclusions and Prospects

The main aims of the present review article are (i) to present recent findings of research concerning neuroprotective/cytoprotective actions of HN, a 24-mer peptide belonging to the MDP family and (ii) to shed further light on the potential of HN to serve as a core template molecule for the eventual development of novel therapeutics, such as suitable HN analogues, against neurodegenerative diseases.

Non-pharmacological interventions and symptomatic treatment with acetylcholinesterase inhibitors (AChEIs) or uncompetitive NMDA receptor antagonists have been the principal forms of fighting AD for more than two decades. Recently, immunotherapy approaches with antitau-protein- or anti-Aβ-based monoclonal antibodies have been developed [97]; two fully human monoclonal antibodies targeting Aβ, i.e., aducanumab [115] and lecanemab [116], have been approved by the FDA, despite some controversy following approval [117]. Nevertheless, due to the widespread prevalence and the severe socioeconomic consequences of AD as well as other neurodegenerative disorders, the development of novel treatment approaches remains of enormous interest. 

New approaches to fight neurodegenerative diseases have been recently reported, including use of peptide vaccination [118] or use of specific peptides capable of targeting neurotoxic aggregates, e.g., of Aβ or α-syn [119,120]. Peptides are increasingly being recognized as important factors involved in key cellular processes. Recent technological breakthroughs have significantly accelerated the development of peptide drugs and, as reported, since 2000, ≥30 non-insulin peptide drugs have been approved for clinical application and ≥170 peptides have been evaluated in clinical trials targeting a broad range of diseases [50,121]. Moreover, pharmacologically active peptides can be chemically synthesized at large scale, in high yield, and at low overall cost, e.g., following specific protocols of solid-phase peptide synthesis [9,50], which is a great advantage supporting further investment in the field. 

HN is an endogenous MDP which was first identified in the occipital lobe of a patient with AD. It is a 24-mer peptide encoded by an sORF of a 16S ribosomal subunit within the mitochondrial genome and each amino acid in the peptide sequence has a specific function in cytoprotective and neuroprotective activities, as demonstrated with several studies. As it was discovered in an AD context, HN seems to have therapeutic potential for fighting pathologies related to AD and other neurodegenerative disorders. However, there is still a lot of research tasks that should be undertaken before eventual exploitation of HN and HN analogues in the fight against neurodegenerative disorders. Thus, full elucidation and understanding of the biological role of HN as a member of the MDP family will further support the attempts to delineate any therapeutic potential of the peptide. Moreover, development and application of highly sensitive and specific methods for HN detection at both the peptide and mRNA levels will certainly help elucidating the biological role of parent HN as well as the mechanisms of action of HN and HN analogues with therapeutic potential, preventing from any non-specific and/or misleading findings. Moreover, the design of novel delivery systems that would facilitate and refine BBB crossing and intracellular release is among the most challenging issues for achieving therapeutic exploitation [36]. Another issue to be addressed is the putative relationship between parent HN and tumorigenesis/metastasis in various types of cancer, which is inconclusive at present [42]; interestingly, the discrepancies between different relevant reports have been attributed, among other factors, to the use of different peptides, e.g., HNG vs. parent HN, since specific HN analogues might interact differently with the receptors present in tumor cells [78]. Based on this, one may speculate that novel HN analogues may be designed, e.g., by employing AI-assisted algorithms, which might prove to interact in a safe and highly desirable way with HN cellular partners, focusing on specific partners in neuronal cells.

In conclusion, HN deserves further investigation as a neuroprotective factor with therapeutic potential against neurodegenerative disorders. To this end, further research is needed aiming at (i) full elucidation of the biological role of parent HN, (ii) development of suitable delivery systems, (iii) design and development of tailor-made HN analogues, i.e., peptide analogues including hybrid/fused HN-based peptides as well as brain-permeable small molecules that function as HN mimetics, with highly desirable physicochemical, biological, and pharmacological characteristics.

## Figures and Tables

**Table 1:** HN and HN analogues with neuroprotective/cytoprotective action.

Code Name	Primary Structure	Evaluation in In Vitro/In Vivo Experimental Models	Ref.
HN	MAPRGFSCLLLLTSEIDLPVKRRA	+/+	[1,2,3]
HNG	MAPRGFSCLLLLT**G**EIDLPVKRRA	+/+	[1,2,3]
HN-D-Ser14	MAPRGFSCLLLLT***s***EIDLPVKRRA	+/−	[21]
HNGF6A	MAPRG**A**SCLLLLT**G**EIDLPVKRRA	+/−	[26]
AGA-(C8R)HNG17	P**A**G**A**S**R**LLLLT**G**EIDLP	+/+	[23]
CL	SALLRSIPAP**A**G**A**S**R**LLLLT**G**EIDLP	+/+	[23]
des-Leu-CL	SALLRSIPAP**A**G**A**S**R**LLL**_**T**G**EIDLP	−/+	[30]
tHN-C3	Hph-1 ^&^-KKRLGLPGDEVDMAPRGFSCLLLLTSEIDLPVKRRA	+/+	[34]
HUJInin	YNAPVSIPQP**A**G**A**S**R**LLLLT**G**EIDLP	+/−	[35]
c[D-Ser14-HN]	c *(MAP**A**G**A**S**R**LLLLT***s***EIDLPVKRRA)	+/−	[35]
HNSS	R(d)-Dmt ^#^-KFGG-MAPRGFSCLLLLT**G**EIDLPVKRRA	+/+	[36]

* *c*: cyclic; ^#^ Dtm: 2′,6′-dimethyl tyrosine; ^&^ Hph-1: human transcription factor containing protein transduction domain (PTD)/cell penetrating peptide (CPP) sequences. All amino acid sequences are presented following the one-letter code. The amino acids that differ between parent HN and each HN analogue appear in red color and bold. Amino acid sequences that are irrelevant to HN appear with a gray background. According to the references shown in the table, HN/HN analogues have been evaluated in in vitro (+/−) or in in vivo (−/+) experimental models, or in both (+/+); moreover, all HN analogues have been prepared with peptide synthesis, except for HNSS which is reported as “commercially available” and tHN-C3, which has been prepared with molecular biology techniques as a fusion peptide.

**Table 2:** Recent and representative in vitro and/or in vivo experimental research findings supporting the therapeutic potential of HN and HN analogues against neurodegenerative/neural/brain disorders and/or pertinent symptoms.

HN/HN Analogue	Disorder/Pathological Symptom Simulated	In Vitro Cellular Model/In Vitro Beneficial Effects	In Vivo Animal Model/In Vivo Beneficial Effects/Main Route of Administration	Ref.
HN	Parkinson’s disease (PD)	SH-SY5Y human neuroblastoma and PC12 rat pheochromocytoma cell lines/ Neuroprotection through mitochondrial biogenesis	Mouse models of PD/Neuroprotection and behavioral recovery/ i.n. administration	[50]
HNSS	Alzheimer’s disease (AD)	HT22 neuronal cells overexpressing FGFR1/Inhibition of experimentally induced intracellular and mitochondrial ROS production	3xTg-AD mice/ Mitochondrial rescue, inhibition of Aβ deposition and tau hyperphosphorylation, amelioration of memory defect and neuronal damage/ i.v. administration through a specially developed delivery system	[36]
HN	ICH	Rat primary cerebral cortical astrocyte and microglia cultures/ Promotion of a “reparative” microglia phenotype characterized by enhanced phagocytosis and reduced proinflammatory responses	C57BL/6J mouse model of ICH induced by intrastriatal injection of autologous blood/ Reduction of neurological deficits, and improvement of hematoma clearance/ i.p. or i.n. administration	[51]
HN	Intracerebral hemorrhage (ICH)	ICH-like cellular model induced in primary rodent neuron cultures/ HN upregulated phosphorylation of STAT3 and increased Mn-SOD expression in neurons under ICH-like injury and prevented ROS-overexpression	C57BL6/J mouse and Sprague Dawley rat models of ICH/ Systemic transplantation of astrocytic mitochondria (which contain HN) in ICH promotes antioxidative protection and assists in functional recovery by enhancing Mn-SOD-mediated neuronal antioxidant defense and neuroplasticity in the brain	[52]
HN	Mild traumatic brain injury (mTBI)	-	Mouse model of mTBI/Reversal of cognitive impairment through restoration of metabolic pathways within astrocytes/ i.p. administration	[53]
HNG	Impairment of object memory	PC12 rat pheochromocytoma cells/ Enhancement of regulated neuronal exocytosis	Normal mice/ Improvement of object memory/ i.p. administration	[54]
HN	Neuronal cell death	Cortical neuron neurotoxicity model induced by calyculin A (CA)/ HN preincubation preserved cell viability, alleviated oxidative stress, blocked tau overphosphorylation, and protected neurons against CA-induced insults	-	[55]
HUJInin and c(D-Ser14-HN)	Stroke	Ischemia–reperfusion injury cellular model induced in PC12 cell cultures using ischemia-like oxygen–glycose-deprivation–reoxygenation insult and SH-SY5Y neuronal cell cultures exposed to pathological H_2_O_2_ oxidative stress/ Cell neuroprotection	-	[35]
HN	Neuronal cell death	SH-SY5Y human neuroblastoma cell line/Neuroprotection against neurotoxicity induced by silver nanoparticles	-	[56]
CL	Stroke	-	C57BL/6 middle cerebral artery occlusion (MCAO) mouse model/ Decrease in the neurological deficits, improvement of motor and cognitive functions, improvement of infarct lesion/ i.p. administration	[57]
HNG	AD	-	APP/PS1 mice/ Improvement of cognitive function/ i.p. administration	[58]
HNG	Age-related cognitive decline	SH-SY5Y cells/ Neuroprotective effect against Aβ mitochondrial toxicity	C57Bl/6N mice/ Improvement of cognitive ability of old-age mice/ i.p. administration	[59]
HNG	Stroke	Mouse brain endothelial cells bEnd.3/ Cytoprotection under oxygen–glucose deprivation (OGD) conditions	Mouse MCAO stroke model/ Amelioration of cerebral infarction and suppression of various inflammatory cytokines/ i.p. administration	[60]

## Data Availability

Not applicable.
